# Challenging Results on FDG PET/CT in a Patient with Uncontrolled Celiac Disease and small bowel adenocarcinoma

**DOI:** 10.22038/AOJNMB.2022.61853.1437

**Published:** 2022

**Authors:** Ali Mohamedkhair, Akram Al-Ibraheem, Ahmed Saad Abdlkadir, Omar Jaber

**Affiliations:** 1 Department of Nuclear Medicine, King Hussein Cancer Center, Amman, Jordan; 2 School of Medicine, The University of Jordan, Amman, Jordan; 3 Department of pathology, King Hussein Cancer Center, Amman, Jordan

**Keywords:** Celiac Disease, FDG PET/CT, Single Short Segmental Bowel Uptake, Adenocarcinoma

## Abstract

Celiac disease (CD) is a chronic immune-mediated enteropathy that is caused by both environmental (gluten) and genetic (human leukocyte antigen (HLA) and non-HLA genes) factors. Patients may be asymptomatic or exhibit atypical symptoms, necessitating a high index of suspicion for proper diagnosis.

The evaluation of CD patients with ^18^F-FDG PET/CT imaging can be difficult, owing to the fact that this disease is inflammatory in nature. Typical ^18^F-FDG PET/CT gastrointestinal manifestations of celiac disease include increased multifocal or diffuse bowel uptake, whereas single short segmental uptake is rarely encountered; thus, awareness of this wide range of findings is important to guide physicians through proper management and outcome.

We report a case of small intestine adenocarcinoma and known CD complaining of recent episodes of diarrhea and weight loss that had a suspicious small bowel wall thickening that corresponds to a short segmental hypermetabolic process on FDG PET/CT follow-up scan. The patient was then referred to the gastroenterology department and underwent a colonoscopy, a biopsy was taken that revealed CD and was negative for malignancy. Furthermore, 6 months later the abovementioned segmental FDG activity was completely resolved without any treatment received at the given time.

## Introduction

 Celiac Disease (CD) is a chronic small intestine immune-mediated enteropathy triggered by exposure to dietary gluten in genetically predisposed individuals (1). Diagnosis of CD is mainly based on serology and histopathological assessment of small intestinal biopsy specimens. Although, sometimes, laboratory and histological findings are inconsistent with patient’s symptoms and insufficient to reach a straightforward diagnosis, it remains the mainstay to detect and assess patients with celiac disease (2). Furthermore, serious complications of CD such as Enteropathy asso-

ciated T-cell lymphoma (EATL), should be considered and ruled out as it is considered the most common neoplastic complication to CD (3). Hoffmann et al, explored the challenges facing ^18^F-FDG PET/CT in diagnosis of EATL in patients with longstanding or untreated CD and concluded that visual and SUV calculations differ significantly between those having ETCL and patients suffering from CD (4). The purpose of this case report is to highlight suspicious findings acquired on ^18^F-FDG PET/CT scan, in a patient with long history of celiac disease who's known to have poor compliance to both diet and therapy. In this light, we'll discuss the challenges 

encountered when interpreting ^18^F-FDG PET/CT scans in such patients, while also shedding the light on the appropriate approaches to follow in order to reach a proper management plan.

## Case report

**Figure 1:**
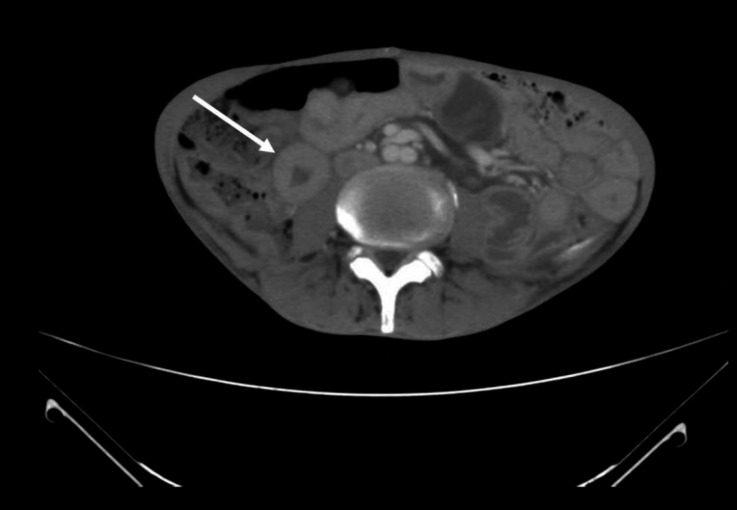
CT scan performed on February of 2021 and showed a questionable circumferential wall thickening (**arrow**) of a distal small bowel loop in the right side of the abdomen which needs further assessment

 A 52-year-old male with known history of uncontrolled celiac disease since 2012. In 2013, he was diagnosed with duodenal adeno-carcinoma after having 2 months history of 

upper abdominal mass. He underwent duoden-ectomy and received chemotherapy upon which he achieved complete remission within the same year. From there, the patient was kept under close follow-up and his condition remained stable until February of 2021 when he begun to complain from persistent diarrhea and weight loss, CT scan of abdomen was done and revealed small intestinal wall thickening involving the ileum (Figure 1). 

**Figure 2:**
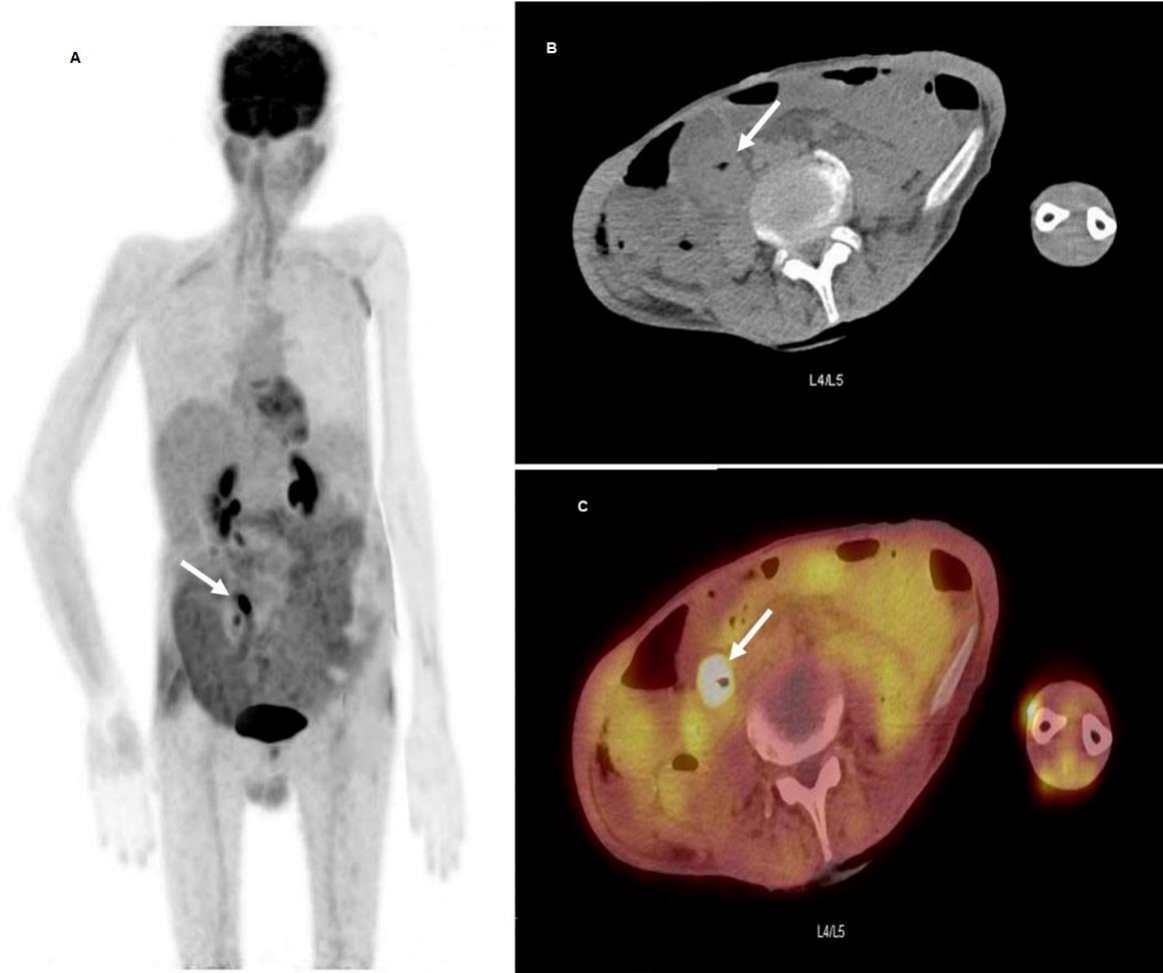
^18^F-FDG PET/CT scan performed on April of 2021 and showed single short segmental hypermetabolic process (**arrows**) within the ileum, manifestation of inflammatory bowel disease vs more serious pathologies such as Enteropathy-associated T cell lymphoma (EATL) or recurrent disease were suspected. Seen on the maximum intensity projection (MIP) image (**A**), as well as on axial CT (**B**) and on axial fused PET/CT (**C**) images at the level of L4/L5 vertebrae

**Figure 3:**
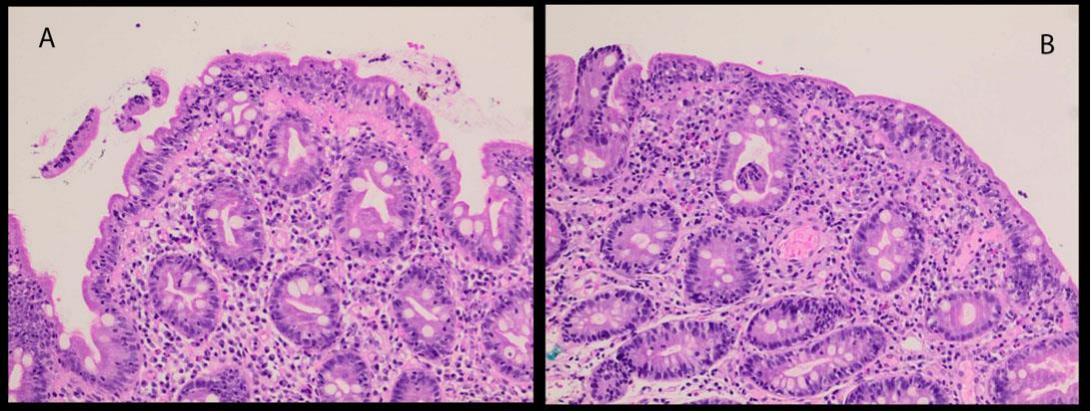
Biopsy specimen of duodenal mucosa performed after baseline FDG PET/CT and showed blunted villi with increased intraepithelial lymphocytes. Hematoxylin and eosin, 400×

**Figure 4:**
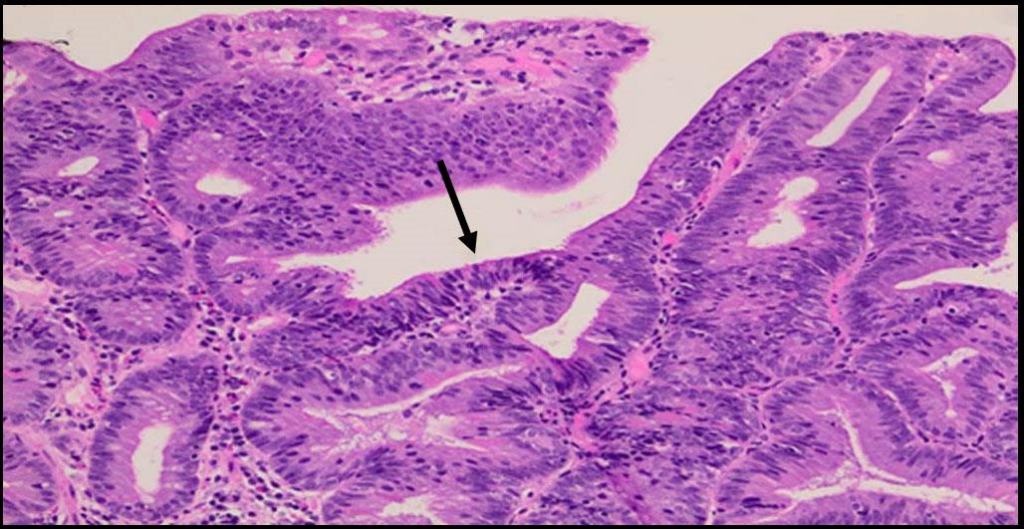
Biopsy specimen of duodenal adenoma showing hyperchromatic pseudostratified adenomatous epithelium reaching the mucosal surface (**arrow**). Hematoxylin and eosin, 400×

**Figure 5:**
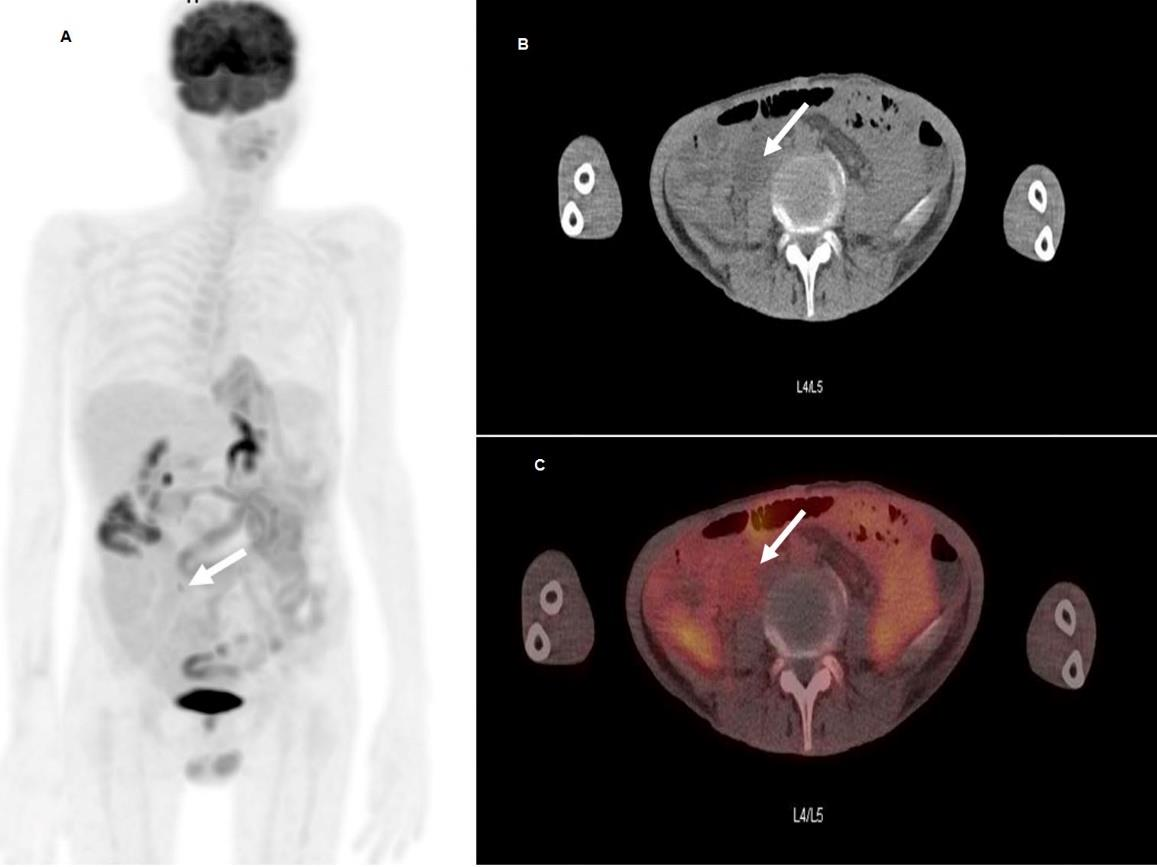
A follow-up ^18^F-FDG PET/CT scan performed 6-month later and showed significant metabolic regression of the previous hypermetabolic segmental lesion (**arrows**) within the small bowel and with no treatment received in the given time, current SUV_max_: 2.5 compared to previous value of 10. Seen on the on the maximum intensity projection (MIP) image (**A**), as well as on axial CT (**B**) and axial fused PET/CT (**C**) images at the level of L4/L5 vertebrae

 Whole-body PET/CT scan was ordered to exclude the presence of a recurrent intestinal tumor/development of EATL vs expected CD features. After IV injection of 177 Mbq ^18^F-FDG dose, images were acquired from the base of the skull to mid of the thighs, PET images reviewed with and without attenuation correction, corresponding CT images (without IV contrast) were used for attenuation correction and anatomical correlation, fasting blood sugar at the injection time was 74 mg/dl and patient weight was 42 Kg. Scan showed a segmental hyper-metabolic process within the small intestine involving the ileum with SUV_max_ of 10 (Figure 2) this was associated with mild diffuse FDG metabolic activity in the remainder of small intestine but with no definite focal lesions. These findings were quite worrisome and required further evaluation by colonoscopy which showed scarring with hyperemia and blunt villi in the terminal ileum and biopsy was taken and confirmed the presence of blunt villous architecture with increased intra-epithelial lymphocyte ratio consistent with celiac disease and no evidence of lymphoma (Figure 3 & 4). 6 months later, another PET/CT scan was performed and showed significant metabolic regression in FDG activity as the SUV_max_ went down from 10, in the previous scan, to 2.5 (Figure 5). This regression occurred while the patient was on follow-up and received no treatment at the given time.

## Discussion

 This case addresses a patient with celiac disease and a history of intestinal adeno-carcinoma, who is known for poor compliance to gluten diet, depicting an atypical, intense, single short segmental FDG activity in the intestine on ^18^F-FDG PET/CT scan which corresponds to an intestinal wall thickening seen on the CT component of the study.

 There are limited available data on ^18^F-FDG

 PET/CT features of CD. Typically, ^18^F-FDG PET/CT scan demonstrates diffuse FDG activity within the affected part of intestine (5). This pattern could be attributed to nature of the disease that’s characterized by inflammatory infiltration of the small intestine. Focal FDG bowel activity, on the other hand is rarely encountered in celiac disease and should warrant further investigation by biopsy to exclude malignancy (6). Thus, it can be quite challenging to interpret cases with focal findings.

 On other hand, CT Imaging is of great importance in assessment of CD. Although findings are diverse, the most common ones include dilation of bowel loops, bowel wall thickening, mesenteric hypervascularity and mesenteric lymphadenopathy (7).

 All references state that ^18^F-FDG PET/CT is of great value in initial diagnosis, disease staging, evaluating response to treatment and restaging of small bowel adenocarcinoma (8). It’s more accurate than CT of abdomen; therefore, it’s of great value and can guide through proper management plans especially in complicated cases of CD that are at great risk to develop malignancy. Furthermore, Muhammed Hadithi et al, conducted a prospective study to assess the role of ^18^F-FDG PET/CT in detection of EATL in patients with refractory CD. Through a sample size of 38 patients he concluded that ^18^F-FDG PET/CT is superior to CT of abdomen in both detection and follow-up of patients with recurrent CD; while any positive PET/CT findings should be assessed by histology and thus will give the clue to proper management plan (9).

 While one cannot deny the clinical importance of ^18^F-FDG PET/CT use in small bowel adenocarcinomas; there are some challenges in PET/CT imaging/reporting in patients with associated infectious or inflammatory disease. As we all know using ^18^F-FDG PET/CT in patients with such scenarios may result in false positive findings that lead to a reduced overall sensitivity (10).

 Eugenia Shmidt et al, who conducted a retrospective cohort study of 41,538 ^18^F-FDG PET/CT scans to assess the clinical significance of incidental FDG uptake in the gastrointestinal tract and discovered that 40% of the patients had suspicious FDG activity only to find out that these lesions are pathologically free via endoscopy; therefore, he concluded that any suspicious focal lesion visualized by ^18^F-FDG PET/CT should be pursued by histopathology to exclude underlying pathology (11).

## Conclusion

 Celiac Disease encompasses a wide spectrum of findings when interpreted by FDG PET/CT. Typically ^18^F-FDG PET/CT demonstrates diffuse or multifocal increased uptake within affected part of small intestine while single focal or short segmental findings are less commonly encountered and may warrant further investigation by biopsy to exclude malignancy. However, interpreting physicians should be aware of the possibility of single short segmental bowel FDG uptake on the background of diffuse mild FDG metabolic bowel activity in patients with celiac disease to avoid misinterpretation and to properly infer the most appropriate recommendations in the PET/CT report.

## Compliance with Ethical Standards

 Funding: The authors received no financial support for the research, authorship or publication of this article.

## Conflict of Interest

 None to declare.

## Ethical approval

 All procedures performed in studies involving human participants were in accordance with the ethical standards of the institutional and/or national research committee and with the 1964 Helsinki declaration and its later amendments or comparable ethical standards.

## Informed Consent

 Informed consent was obtained from the patient for publication of his case/report and accompanying images.
